# miR-874 functions as a tumor suppressor by inhibiting angiogenesis through STAT3/VEGF-A pathway in gastric cancer

**DOI:** 10.18632/oncotarget.2748

**Published:** 2015-01-22

**Authors:** Xiaoyu Zhang, Jie Tang, Xiaofei Zhi, Kunling Xie, Weizhi Wang, Zheng Li, Yi Zhu, Li Yang, Hao Xu, Zekuan Xu

**Affiliations:** ^1^ Department of General Surgery, The First Affiliated Hospital of Nanjing Medical University, Nanjing, Jiangsu, China; ^2^ Division of Gastrointestinal Surgery, Department of General Surgery, Huai'an People's Hospital, Xuzhou Medical College, Huai'an, Jiangsu, China

**Keywords:** microRNA-874, tumor angiogenesis, STAT3, VEGF-A, gastric cancer

## Abstract

MicroRNAs are endogenously expressed, small non-coding RNAs that regulate gene expression by targeting mRNAs for translational repression or degradation. Our previous studies indicated that miR-874 played a suppressive role in gastric cancer (GC) development and progression. However, the role of miR-874 in tumor angiogenesis and the mechanisms underlying its function in GC remained to be clarified. Here, gain- and loss-of-function assays demonstrated that miR-874 inhibited the tumor angiogenesis of GC cells *in vitro* and *in vivo*. Through reporter gene and western blot assays, STAT3 was shown to be a direct target of miR-874. Overexpression of STAT3 rescued the loss of tumor angiogenesis caused by miR-874. Conversely, the STAT3-shRNA attenuated the increased tumor angiogenesis caused by the miR-874-inhibitor. Furthermore, the levels of miR-874 were inversely correlated with those of STAT3 protein in GC tissues. Taken together, these findings indicate that down-regulation of miR-874 contributes to tumor angiogenesis through STAT3 in GC, highlighting the potential of miR-874 as a target for human GC therapy.

## INTRODUCTION

Gastric cancer (GC) is the fourth most prevalent type of malignancy worldwide, and it is the second most frequent cause of death from cancer [1]. Despite significant achievements in the treatment of early GC, the long-term survival rate for advanced GC remains quite low [2]. The five-year survival rate for advanced or metastatic gastric cancer is only 5–20%, with a median overall survival of less than 1 year [3, 4]. Consequently, the molecular mechanisms that regulate GC development and progression need further exploration.

Angiogenesis is vital for tumorigenesis and development, as tumors cannot grow larger than 2 mm in diameter without angiogenesis. Recent studies have shown that several miRNAs are involved in the regulation of vascular development [5,6,7]. miRNA-378 promotes tumor angiogenesis by targeting Sufu and Fus-1 [8]. miRNA-132 acts as an angiogenic switch by suppressing endothelial p120RasGAP expression, leading to Ras activation and the induction of neovascularization [9]. Conversely, miR-15b, miR-16, miR-20a and miR-20b are potential anti-angiomiRs that may function through targeting VEGF [10]. In a previous study, we demonstrated that miR-874 suppressed the growth, migration and invasion of gastric cancer cells [11]. We also observed that xenografted tumors from pre-miR-874-transfected cells were smaller and had a lower microvessel density (MVD) than tumors from miR-874-inhibitor-transfected cells. However, the exact mechanisms underlying the regulation of angiogenesis in GC by miR-874 remain unknown. Using bioinformatics, we identified STAT3 – a key transcription factor [12] that plays a vital role in human gastric cancer angiogenesis [13] – as a potential direct target of miR-874. Therefore, we investigated the role of miR-874 in GC angiogenesis and its relationship with the STAT3 pathway.

In this study, we found that miR-874 strongly repressed GC angiogenesis by targeting the 3′ untranslated region (3′-UTR) of the STAT3 mRNA, leading to inhibition of the STAT3 pathway and down-regulation of the angiogenic factor VEGF-A. Our results suggest that down-regulation of miR-874 may be important for the development and progression of GC, highlighting the potential for miR-874 as a therapeutic target.

## RESULTS

### miR-874 is down-regulated in human gastric cancer tissues and cells

**Figure 1:**
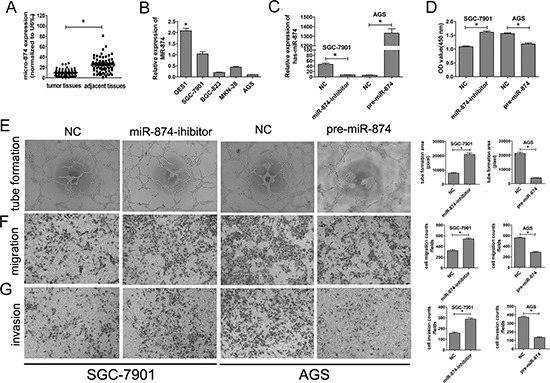
The expression of miR-874 in GC tissues and cell lines. miR-874 negatively regulates HUVEC proliferation, migration, invasion and tube formation *in vitro* **(A)** Tissue samples from eighty paired adjacent normal tissues and GC tissues were used to extract total RNA. The levels of miR-874 were analyzed by miRNA RT-PCR. U6 was used as an internal control. **(B)** The expression levels of miR-874 were analyzed by miRNA RT-PCR in a normal gastric mucosa epithelial cell line (GES-1) and the GC cell lines AGS, BGC823, MKN28 and SGC-7901. **(C)** SGC-7901 or AGS cells were transfected with a specific miR-874 inhibitor, pre-miR-874 or empty lentiviral construct vectors. miRNA RT-PCR was used to analyze the expression levels of miR-874. **(D)** The OD value of the HUVECs (determined by CCK8 assay) were used to calculate the number of cells for each group(SGC-7901-NC, SGC-7901-miR-874-inhibitor, AGS-NC and AGS-pre-miR-874). **(E)** Tube-formation assays with HUVECs were performed with the different group of conditioned medium. The network area was calculated using Image Pro Plus 6. **(F–G)** Transwell migration and Matrigel invasion assays with HUVECs were performed in each group. Asterisks indicate a significant difference compared with controls at *P* < 0.05.

To determine whether miR-874 is mis-regulated in GC tissues, miR-874 expression in GC tissues and adjacent normal tissues was analyzed using miRNA RT-PCR. As shown in Fig. 1A, the expression levels of miR-874 in human GC tissues were much lower than in the adjacent normal tissues. Further experiments showed that several GC cell lines, including AGS, BGC823, MKN28 and SGC7901, had undetectable or low levels of miR-874. By contrast, normal gastric mucosa epithelial cells (GES-1) had high levels of miR-874 (Fig. 1B).

These results indicate that miR-874 is down-regulated in both GC tissues and cancer cell lines. To investigate the impact of miR-874 on tumor angiogenesis, we constructed both miR-874 overexpression and knockdown GC cell lines (Fig. 1C). As shown, miR-874 was knockdown about 80% in SGC7901 cells, increased about 140 folds in AGS cells.

### miR-874 regulates the processes of tumor angiogenesis *in vitro*

To confirm that miR-874 is a potential angiogenesis suppressor in GC, we investigated the influence of miR-874 on the tube formation, proliferation, migration and invasion of HUVEC cells *in vitro*.

Tube-formation assays with HUVECs were performed with the different group of conditioned medium (miR-874 inhibitor, pre-miR-874 or empty vector). Compared with control cells, the silencing of miR-874 group increased the tube-forming capacity of HUVECs, whereas ectopic expression of miR-874 group dramatically reduced the tube-forming capacity of HUVECs (Fig. 1D).

Next, we used cell migration and Matrigel invasion assays to investigate the effects of miR-874 on HUVEC migration and invasion. Our data revealed that HUVECs migration was enhanced by miR-874 knockdown in SGC-7901 cells, whereas migration was suppressed by miR-874 overexpression in AGS cells (Fig. 1E). Additionally, compared with control cells, the silencing of miR-874 in SGC-7901 cells dramatically boosted HUVECs invasiveness, whereas miR-874 up-regulation inhibited HUVECs invasiveness, as assessed using a Matrigel invasion assay (Fig. 1F).

We also used the CCK8 assay to assess the effects of miR-874 on HUVECs proliferation. The proliferation of HUVECs in the miR-874 inhibitor-treated group was significantly increased compared with the control group in SGC-7901 cells. By contrast, the conditioned medium of AGS cells transfected with the miR-874 precursor caused a significant decrease in HUVECs proliferation compared with the negative control group (Fig. 1G).

### VEGF-A expression is inhibited by miR-874

**Figure 2:**
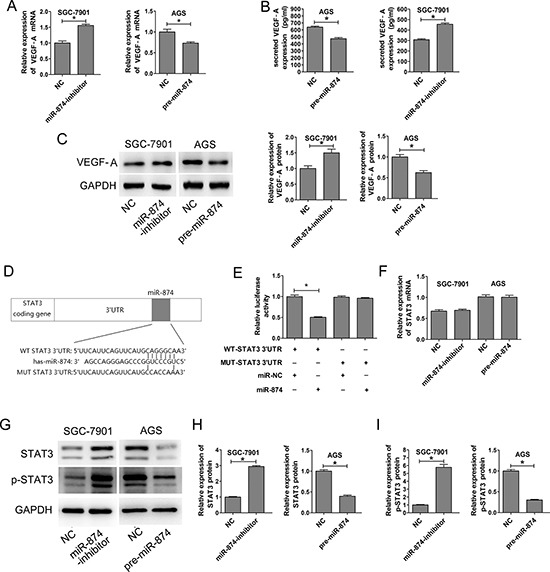
miR-874 inhibits VEGF expression. Identification of STAT3 as a potential target of miR-874 **(A)** qRT-PCR was used to analyze the expression levels of VEGF-A in SGC-7901 and AGS cells transfected with miR-NC, the miR-874 inhibitor or pre-miR-874. **(B)** ELISA was used to determine the expression of VEGF-A in the culture medium of SGC-7901 and AGS cells transfected with miR-NC, the miR-874 inhibitor or pre-miR-874. **(C)** VEGF-A protein levels were analyzed by Western blotting analysis. **(D)** STAT3 3′-UTR regions containing the wild-type or mutant binding site and the sequence complementarity between miR-874 and the STAT3 3′-UTR (WT and MUT) are shown. **(E)** Relative STAT3 luciferase activity was analyzed after co-transfection with the wild-type or mutant 3′-UTR reporter plasmids. The histogram represents the mean normalized 3′-UTR luciferase intensity from three independent experiments (mean ± s.d.). **(F)** qRT-PCR was used to analyze the mRNA levels of STAT3 in SGC7901 and AGS cells transfected with miR-NC, miR-874 inhibitor or pre-miR-874. **(G–I)** Western blotting assays were used to analyze the expression levels of STAT3 and p-STAT3 in SGC-7901 and AGS cells transfected with miR-NC, miR-874 inhibitor or pre-miR-874. Relative densities were quantified using Image-Pro Plus software. The results are presented as mean ± s.d. values from three duplicate experiments. Asterisks indicates significant differences compared with controls at *P* < 0.05.

SGC-7901-NC, SGC-7901-miR-874-inhibitor, AGS-NC and AGS-pre-miR-874 cells were used to test the levels of VEGF-A, which is the most important angiogenic factor influencing vasculature and angiogenesis. As shown in Fig. 2A, when compared with SGC7901-NC cells, VEGF-A mRNA levels in the SGC-7901-miR-874-inhibitor cells were increased by approximately 33%. Compared with the AGS-NC cells, VEGF-A mRNA levels in the AGS-pre-miR-874 cells were decreased by approximately 30%. Similarly, the protein levels of VEGF-A were increased in SGC-7901-miR-874-inhibitor cells compared with SGC7901-NC cells, and they were also decreased in AGS-pre-miR-874 cells compared with AGS-NC cells (Fig. 2B). We also used ELISA to detect secreted VEGF-A protein in the supernatants of the above cell lines. As expected, knockdown of miR-874 increased secreted VEGF-A protein expression, and overexpression of miR-874 decreased secreted VEGF-A protein expression (Fig. 2C).

### The 3′-UTR of STAT3 is a target of miR-874

TargetScan (http://www.targetscan.org/), PicTar (http://pictar.bio.nyu.edu), miRanda (http://www.sanger.ac.uk), and miRBase (http://www.mirbase.org) were used to predict genes which miR-874 might target. We identified a putative miR-874 binding site within the 3′-UTR of *STAT3* (Fig. 2D), and a luciferase reporter assay was used to validate whether STAT3 is a direct target of miR-874. Wild-type (WT) and mutant (MUT) versions of the STAT3 3′-UTR – the latter containing site-directed mutations in the putative miR-874 target sites – were cloned into reporter plasmids. Forced expression of miR-874 markedly suppressed luciferase activity from the wild-type reporter (50%) but not from the mutant reporter, suggesting that the 3′-UTR of *STAT3* is targeted by miR-874 and that the point mutations in this sequence abolished this effect (Fig. 2E).

### miR-874 suppresses STAT3 protein expression through translational repression

miR-874 silencing in SGC7901 cells, which lack endogenous STAT3 expression, resulted in the up-regulation of STAT3 protein by approximately 3 folds compared with the negative control. Conversely, the protein levels of STAT3 were significantly reduced about 67% in AGS cells, which exhibit basally high expression of STAT3, after transfection with pre-miR-874 (Figs. 2G and 2H). In addition, the activated form of STAT3 (p-STAT3, Tyr705) was significantly increased in miR-874 knockdown cells (SGC-7901) and decreased in miR-874-overexpressing cells (AGS) (Figs. 2G and 2I). In contrast, no significant changes were observed for STAT3 mRNA levels (Fig. 2F). These results indicate that miR-874 suppresses STAT3 protein expression through translational repression.

### miR-874 inhibits tumor growth, angiogenesis *in vivo* and negative correlated with STAT3, VEGF-A expression

**Figure 3:**
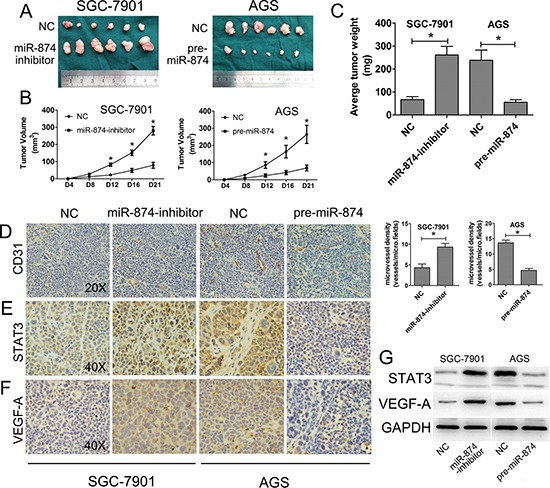
miR-874 inhibits tumorigenicity *in vivo* **(A)** Photographs of tumors derived from the different groups of nude mice. **(B–C)** The graph is representative of tumor growth 21 days after inoculation. Tumor volume and the weight were calculated, and all date are shown as mean ± s.d. **(D–F)** The expression of CD31, VEGF-A and STAT3 were analyzed in tumor tissues by immunohistochemistry; representative images are shown at magnification × 20 (CD31) or × 40 (STAT3, VEGF-A). Blood vessels were stained using anti-CD31 antibodies, and positively stained blood vessels were counted in five areas per slide to determine the maximum number of microvessels; 10 slides per experiment. The results are presented as mean ± s.d. values (*n* = 10). **(G)** The relative expression of STAT3 and VEGF-A protein in the different groups were analyzed by western blotting. Asterisks indicate significant differences compared with negative controls versus SGC-7901-miR-874 inhibitor and AGS-pre-miR-874, respectively; *P* < 0.05.

To determine the effects of miR-874 on tumorigenicity *in vivo*. Transfected cells were injected into the flanks of nude mice to form ectopic tumors. After 21 days, we observed a slower tumor growth in the miR-874-NC group than in the miR-874-inhibitor group (SGC-7901 cells). Similar phenomenon was observed in AGS cells, that tumor in pre-miR-874 group grow slower than in miR-874-NC group (Figs. 3A, 3B and Supplementary Fig. 1). The average weight of tumors from the miR-874-inbibitor group was significantly more than that from the control group in SGC-7901 cells. In AGS cells, we could find that the tumors from pre-miR-874 group were heavier than that from the control group, which was consistent with the results in SGC-7901 cells (Fig. 3C). Moreover, the immunohistochemical assays showed that the number of CD31-positive microvessels was dramatically increased about 3 folds by the SGC-7901-miR-874 inhibitor, whereas AGS-pre-miR-874 decreased the number of CD31-positive microvessels to 65% (Figs. 3D). Similar trends were observed with respect to the expression of STAT3 and VEGF-A in the tumors. (Figs. 3E and 3F). Furthermore, western blotting analysis of the implanted mouse tumors verified that STAT3 and VEGF-A protein expression were significantly enhanced in the SGC-7901-miR-874 inhibitor-transfected group compared with the controls, whereas their expression were decreased in the AGS-pre-miR-874-transfected group (Fig. 3G). Taken together, we found that miR-874 could inhibit tumor growth and angiogenesis *in vivo*, and that the negative correlation between miR-874 expression and STAT3 or VEGF-A levels.

### miR-874 inhibits tumor angiogenesis by targeting STAT3

**Figure 4:**
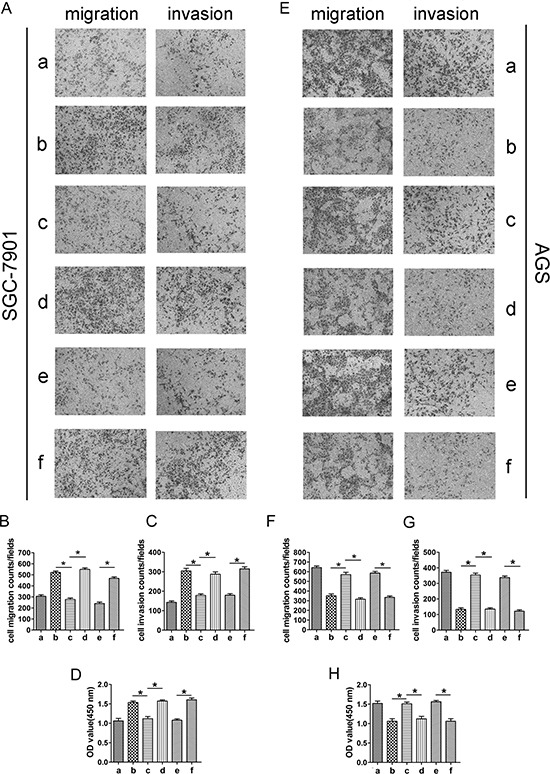
The roles of miR-874 and STAT3 in the regulation of HUVEC proliferation, migration and invasion SGC-7901(a: miR-874-NC; b: miR-874-inhibitor; c: miR-874-inhibitor+STAT3-shRNA; d: miR-874-inhibitor+STAT3-shcontrol; e: LV-NC; f: LV-STAT3). AGS (a: miR-874-NC; b: pre-miR-874; c: pre-miR-874+LV-STAT3; d: pre-miR-874+LV-NC; e: STAT3-shcontrol; f: STAT3-shRNA). **(A–H)** HUVECs cultured with different conditioned media were subjected to CCK8 assays, as well as cell migration and invasion assays. The reverse experiments for miR-874 inhibitor/pre-miR-874 were performed through the down-regulation/overexpression of STAT3. Representative data are displayed as mean ± SD values. Asterisks indicate significant differences when compared with controls at *P* < 0.05.

**Figure 5:**
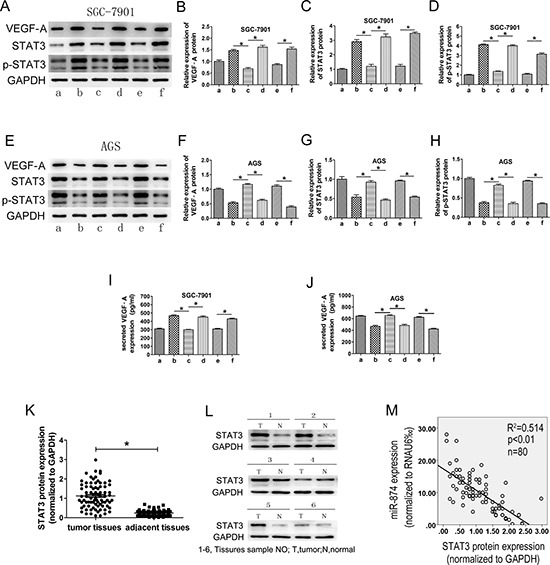
The roles of miR-874 and STAT3 in the regulation of VEGF-A expression in gastric cancer cellsSTAT3 protein levels were negatively correlated with miR-874 levels in GC tissues SGC-7901 (a: miR-874-NC; b: miR-874-inhibitor; c: miR-874-inhibitor+STAT3-shRNA; d: miR-874-inhibitor+STAT3-shcontrol; e: LV-NC; f: LV-STAT3). AGS (a: miR-874-NC; b: pre-miR-874; c: pre-miR-874+LV-STAT3; d: pre-miR-874+LV-NC; e: STAT3-shcontrol; f: STAT3-shRNA). **(A–D, E–H)** Western blotting analysis was used to detect the expression of VEGF-A, STAT3 and p-STAT3. **(I–J)** ELISA was used to test the levels of VEGF-A in the supernatants. Data were collected from at least three independent experiments. **(K)** The expression of STAT3 in human GC specimens and adjacent normal tissues was assayed by Western blotting analysis. STAT3 expression was normalized to GAPDH levels. STAT3 expression levels in GC tissues and adjacent normal tissues were measured and analyzed, as shown by the scatterplot. **(L)** Representative immunoblotting results from 6 pairs of GC tissues and adjacent noncancerous tissues. **(M)** Linear correlation analysis was used to determine the correlation between the expression levels of STAT3 and miR-874 using SPSS software (*p* < 0.01). A representative data set is displayed as mean ± SD values. Asterisks indicate significant differences when compared with controls at *P* < 0.05.

We demonstrated that ectopic expression of miR-874 in GC cells suppressed the tube formation, proliferation, migration and invasion of HUVECs and inhibited VEGF-A and STAT3 protein expression. By contrast, miR-874 knockdown promoted these behaviors and enhanced VEGF-A and STAT3 protein expression (Figs. 1, 2, 3). To further demonstrate that miR-874 in GC cells affects the angiogenesis of HUVECs through the regulation of STAT3, we up-regulated and down-regulated STAT3 expression. The results showed that that enhanced expression of STAT3 in GC cells (SGC-7901) promoted the proliferation, migration and invasion of HUVECs (Figs. 4A–D; e, LV-NC *vs* f, LV-STAT3), whereas knockdown of endogenous STAT3 (AGS) inhibited these behaviors in HUVECs (Figs. 4E–4H; e, STAT3-shcontrol *vs* STAT3-shRNA). Intriguingly, the inhibitory effect of STAT3 silencing on these cellular phenotypes was consistent with the effect of miR-874 overexpression. Subsequently, we investigated whether STAT3 could counteract the suppression of these cellular phenotypes induced by miR-874 overexpression in HUVECs. The vector LV-STAT3, which contains only the STAT3 coding sequence, was constructed for STAT3 expression without miR-874 targeting. AGS cells were co-transfected with miR-874 precursor and either LV-STAT3 or LV-NC. The data clearly confirmed that ectopic expression of STAT3 effectively reversed the suppression of HUVEC proliferation, migration and invasion caused by miR-874 overexpression (Figs. 4E–H; c, pre-miR-874+LV-STAT3 *vs* d, pre-miR-874+LV-NC). In SGC-7901 cells, we observed a similar phenomenon, which could be counteracted by down-regulation of STAT3 (Figs. 4A–D; c, miR-874-inhibitor+STAT3-shRNA *vs* d, miR-874-inhibitor+STAT3-shcontrol). In addition, we observed similar trends when we tested the levels of VEGF-A, STAT3, p-STAT3 protein and secreted VEGF-A protein in the supernatant (Figs. 5A–J). Taken together, these results confirmed our hypothesis that miR-874 in GC cells affects HUVEC proliferation, migration, invasion and VEGF-A expression by regulating STAT3 expression.

### miR-874 expression is negatively correlated with the STAT3 levels in human GC tissues

To determine whether reduced miR-874 expression correlates with increased levels of STAT3 in GC tissues, eighty pairs of primary GC tissues and adjacent normal tissues were used to determine the STAT3 expression using Western blotting analysis. The results indicated that STAT3 protein levels in GC tissues were dramatically higher than in adjacent normal tissues (Figs. 5K and 5L). As shown in Fig. 5M, linear correlation analysis showed a significant inverse correlation between miR-874 and STAT3 expression in GC tissues (*P* < 0.01), confirming that decreased expression of miR-874 was significantly associated with increased STAT3 protein expression in this set of GC tissues.

## DISCUSSION

miRNAs are short (20–24 nt), stable, non-coding RNA molecules that regulate 60% of coding genes by binding to mRNA molecules to prevent translation and/or promote degradation. To date, over 1,000 miRNAs have been identified, and they have been shown to participate in nearly all biological processes, including cell proliferation and tumor angiogenesis. Indeed, novel functions and mechanisms by which miRNAs regulate their target genes are regularly discovered [14, 15].

Many miRNAs have been shown to act as either oncogenic factors or tumor suppressors, with their specific functions depending on the targeted mRNA. Activation of oncomiRNAs leads to inhibition of tumor suppressor genes, facilitating cell proliferation and tumor progression. Conversely, the decreased activity of tumor-suppressor miRNAs leads to increased oncogene translation, contributing to tumor formation [16].

miR-874 has been identified as a tumor-suppressor and is reportedly down-regulated in some types of cancer, including GC [17–21]. Interestingly, mir-874 is also involved in Mild Cognitive Impairment (MCI), such as Alzheimer's diseases [22]. In the present study, we confirmed that miR-874 expression is significantly lower in GC tissues and cell lines. These results indicate that the down-regulation of miR-874 plays an important role in the initiation and development of GC.

In our previous study, we demonstrated that miR-874 plays a suppressive role in the growth, migration and invasiveness of GC cells [11]. In addition to these behaviors, tumor angiogenesis is also important for tumor progression. Angiogenesis is the process by which new micro-vessels sprout from pre-existing blood vessels. Abundant neovascularization is necessary for adequate nutrition during tumor development, including metastasis. Recent studies have shown that miRNAs (e.g., miR-26a, miR-103, miR-125b, miR-132, and miR-107) regulate endothelial cell functions and affect blood vessel formation and extension [9, 23–26]. Therefore, we hypothesized that miR-874 may contribute to tumor angiogenesis in GC.

Tumor angiogenesis is crucially dependent on communication between the tumor and the associated endothelium [27]. The migration, invasion, proliferation and tube formation of endothelial cells (ECs) are important processes for tumor angiogenesis [28]. Here, we describe a role for miR-874 in inhibiting angiogenesis, which is supported by a number of *in vitro* and *in vivo* experiments. miR-874 depletion in GC cells promotes HUVEC proliferation, migration, invasion and tube formation *in vitro* and increases micro-vessel density *in vivo*. By contrast, enhanced expression of miR-874 suppressed these effects. Further experiments revealed that miR-874 could attenuate tumor angiogenesis by down-regulating expression of VEGF-A. These results strongly suggest that down-regulation of miR-874 enhances the development and progression of GC.

However, the mechanisms underlying how miR-874 affects tumor angiogenesis were not clear. Therefore, we searched for potential targets of miR-874 in gastric cancer cells using several computational algorithms. Among the candidate target genes, we focused on STAT3 because of its known role as a regulator of many critical functions in both normal and malignant human tissues, including angiogenesis, differentiation, proliferation, survival, and immune functions [29–31]. Constitutive STAT3 activation promote VEGF-A expression and stimulates tumor angiogenesis [13, 32, 33]. Our study shows that miR-874 negatively regulates STAT3 at the post-transcriptional level by binding to a specific target site within the 3′-UTR. Overexpression of miR-874 in human GC cell lines inhibited STAT3 and p-STAT3 production at the translational level, and ectopic expression of STAT3 effectively reversed the suppression of HUVEC proliferation, migration, invasion and VEGF-A expression caused by miR-874 overexpression. In addition, a STAT3 shRNA impaired the enhanced angiogenesis caused by miR-874 knockdown. These results suggest that the inhibitory effects of miR-874 on angiogenesis are dependent on STAT3.

**Figure 6:**
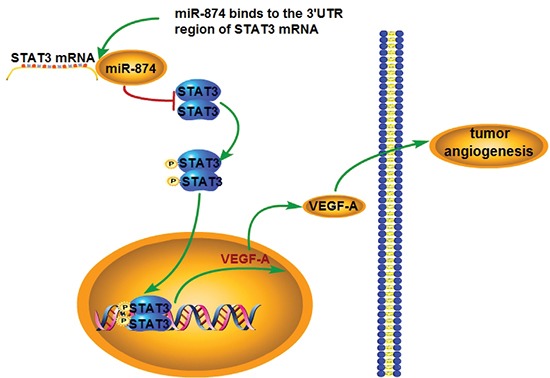
Schematic representation of the roles of miR-874 on the angiogenic properties of GC cells miR-874 suppresses STAT3 (p-STAT3) protein expression by binding to the 3′region of STAT3 mRNA. The decreased expression of STAT3 (p-STAT3), in turn leads to reduce the expression of VEGF-A. Thus, the angiogenic properties of GC cells was enhanced.

We do note that this study has certain limitations. Our results showed that miR-874 can inhibit angiogenesis through the STAT3/VEGF-A pathway (Fig. 6). However, the regulation of angiogenesis-related cytokines in cancer cells is quite complex, and we do not rule out the possibility that other signaling pathways that modulate VEGF-A expression may also be affected by miR-874.

In summary, we present evidence that miR-874 suppresses GC progression by modulating angiogenesis through the STAT3/VEGF-A pathway. We also demonstrate that the levels of miR-874 expression in resected patient gastric tumor tissues are significantly lower than in adjacent normal tissues and that they are inversely correlated with STAT3 protein levels in these tumors. These findings indicate that our study has clinical relevance and that miR-874 overexpression and/or strategies for inhibiting STAT3/VEGF-A signaling may have therapeutic applications for gastric cancer.

## MATERIALS AND METHODS

### Tissue samples

Written informed consent was obtained prior to collection. Acquisition of tissue specimens and the study protocol were performed in strict accordance with the regulations of the Nanjing Medical University Institutional Review Board. Paired specimens of human gastric cancer tissue and adjacent normal gastric tissue were obtained from 80 patients with GC who had undergone surgical operations at the Department of General Surgery, First Affiliated Hospital, Nanjing Medical University, China, and samples were immediately frozen in liquid nitrogen until RNA and protein extraction. In all cases, diagnoses and grading were confirmed by two experienced pathologists and were carried out in accordance with the Cancer criteria of the American Joint Committee.

### Cells and cell culture

The human GC cell lines AGS(ATCC, USA) and BGC823, MKN28, SGC-7901 as well as the human normal gastric epithelial cell line GES-1 (CBTCCCAS, China) were cultured in RPMI-1640 medium supplemented with 10% fetal bovine serum (WISENT, Canada) and antibiotics (1% penicillin/streptomycin, Gibco, USA). Human umbilical venous endothelial cells (HUVECs) (ATCC, USA) were cultured in Endothelial Cell Growth Medium. All cell lines were grown in a humidified chamber supplemented with 5% CO_2_ at 37°C.

### Quantitative polymerase chain reaction (qRT-PCR)

Total RNA was extracted from frozen tissues and cell lines using Trizol Reagent (Invitrogen, USA), according to the manufacturer's protocol, and reverse transcribed into cDNA using Primescript RT Reagent (Takara, Japan). qRT-PCR was performed using a 7500 Real-Time PCR System (Applied Biosystems, USA) with Fast Start Universal SYBR Green Master (Roche, USA). The specific primers were as follows: STAT3, forward: 5′-CAGCAGCTTGACACACGGTA-3′, reverse: 5′-ACACCAAAGTGGCATGTGA-3′; VEGF-A, forward: 5′-CCTGGTGGACATCTTCCAGGAGTACC-3′, reverse: 5′-GAAGCTCATCTCTCCTATGTGCTGGC-3′; and β-actin, forward: 5′-AGAAAATCTGGCACCACACC-3′, reverse: 5′-TAGCACAGCCTGGATAGCAA-3′. All procedures were performed in triplicate.

### miRNA RT–PCR

Total RNA was extracted as above. Target-specific reverse transcription and Taqman microRNA assay probes were assayed using the Hairpin-it™ miRNA qPCR Quantitation Kit (Genepharma, CHINA), according to the manufacturer's instructions. The reactions were also performed using the 7500 Real-Time PCR System. The snRNA U6 was selected as an endogenous reference to calculate the relative expression levels of miR-874 in each sample using the 2^−ΔΔCt^ method. All experiments were performed independently in triplicate.

### Vector constructs, lentivirus production and cell transfections

We modified the commercial LV3-has-miR-874-pre-microRNA vector (pre-miR-874) and LV3-has-miR-874-sponge inhibitor vector (miR-874 inhibitor) lentiviral constructs (Genepharma, CHINA). An LV3 scrambled lentiviral construct (miR-NC) was used as a negative control. All vectors were verified by DNA sequencing. The miR-874-NC, pre-miR-874 and miR-874 inhibitor lentiviral vectors were used to infect cells at an appropriate multiplicity of infection (MOI) when AGS and SGC7901 cells had grown to 40–50% confluence. SGC-7901-NC, SGC-7901-miR-874 inhibitor, AGS-NC and AGS-pre-miR-874 stable cell lines were selected for using 3 μg/ml bulk puromycin-resistant culturing (puromycin, Sigma, USA) for five days. After that, cells were analyzed for miR-874 expression using the Hairpin-it™ miRNA qPCR Quantitation Kit.

Vectors containing green fluorescent protein (GFP) and the puromycin sequence for the overexpression and shRNA targeting of human STAT3 using lentiviral gene transfer were constructed by Genephama Biotech (Shanghai, China). The scrambled lentiviral construct was used as a negative control. For the shRNA targeting of human STAT3, we used the oligonucleotide sequence GCGTCCAGTTCACTACTAA. When SGC7901 cells were at 40–50% confluence, the cells were transfected with the lentiviral vectors (LV-STAT3, LV-NC; STAT3-shRNA, STAT3-shcontrol). Stable cell lines were selected using 3 μg/ml bulk puromycin-resistant cultures (puromycin, Sigma, USA) for one week. Afterward, cells were analyzed using quantitative RT-PCR and Western blotting analysis for STAT3 expression.

### 3′-UTR luciferase constructs and assay

The 3′UTR of the *STAT3* mRNA containing either the putative or mutated miR-874 binding site was synthesized by Shenggong (Shanghai, China). This sequence was cloned into the FseI and XbaI restriction sites of the pGL3 luciferase control reporter vector (Promega, USA) to generate the *STAT3* 3′UTR reporter. A total of 5 × 10^5^ AGS cells stably transfected with pre-miR- 874 or miR-NC were seeded into 24-well plates. Cells were cotransfected with 0.12 μg pGL3-WT-STAT3 or pGL3-MUT-STAT3 3′UTR reporter plasmid. In addition, 0.01 μg Renilla luciferase expression plasmid was cotransfected into the above cells as a reference control. Firefly and Renilla luciferase activities were measured using the Dual-Luciferase reporter assay (Promega, USA) 36 h after transfection, according to the manufacturer's instructions. Relative luciferase activity was calculated as the ratio of firefly fluorescence/Renilla fluorescence.

### Western blotting assay

Total protein from frozen tissue and cell lines was extracted using the following lysis buffer: 50 mM Tris–HCl (pH 7.4), 150 mM NaCl, 1% Triton X-100, 0.1% SDS, 1 mM EDTA, and protease inhibitors (1 mM phenylmethanesulfonyl fluoride and a protease inhibitor cocktail). The protein extracts were size-fractionated using SDS–polyacrylamide gel electrophoresis and transferred to PVDF membranes (Bio-Rad, USA). After blocking, the membranes were incubated with specific primary antibodies in dilution buffer at 4°C overnight. The blotted membranes were incubated with HRP-conjugated anti-mouse or anti-rabbit IgG (Biotime, China) antibodies at room temperature for 2 h. Next, protein expression levels were detected using an enhanced chemiluminescence (ECL) detection system, according to the manufacturer's instructions. GAPDH was used as an internal control. Mouse antibodies to STAT3, rabbit antibodies to p-STAT3 (Cell Signaling Technology, USA), and mouse antibodies to VEGF-A(Abcam, UK) were used.

### ELISA for VEGF-A

The protein levels of VEGF-A in the supernatant were measured using the Quantikine human VEGF-A ELISA kit (NeoBioscience, China), according to the manufacturer's instructions. Briefly, cells were seeded into 6-well plates and cultured to 80% confluence, and the cells were then switched to fresh medium. The supernatants were collected, and the number of cells in each well was counted after 24 h. The level of VEGF-A in the supernatant (100 μl) was determined and normalized to the cell number. A serial dilution of human recombinant VEGF-A was included in each assay to create a standard curve.

### HUVEC proliferation assay

SGC-7901 and AGS cells were cultured as described above. When the cells reached 80% confluence, the culture medium was changed to RMPI-1640 without fetal bovine serum. Following an additional 24 h of culturing, the supernatant was collected as conditioned medium and stored at –80°C. HUVECs were suspended at a density of 2 × 10^4^ cells/ml, and 100 μl cells per well were seeded into 96-well plates. After 24 h, the medium was changed to conditioned medium, as described above. Cell proliferation was assessed using the Cell Counting Kit-8 (Dojindo Laboratories, Japan), according to the manufacturer's protocol. At time point 72 h, 10 μl of cell proliferation reagent solution was added to each well of the 96-well plate, and the cells were incubated for 3 h in a CO_2_ incubator. The absorbance at 450 nm (OD value) was measured using a microplate reader; absorbance at 630 nm was used as a reference. Average OD values were used to calculate the total number of cells for each group.

### HUVEC migration and invasion assays

Cell migration and invasion assays were performed using a chamber 6.5 mm in diameter with an 8 μm pore size (Corning, USA). The upper chambers were seeded with 1 × 10^4^ HUVEC cells. Subsequently, the different groups of conditioned medium were added to the lower chamber. For the invasion assay, the top chamber was coated with 100 μl of 1 mg/ml Matrigel (BD, USA). Cells were incubated at 37°C for 36 h, and then cells in the upper chamber were removed using cotton swabs. Cells migrating into or invading the bottom of the membrane were stained with 0.1% crystal violet for 20 min at 37°C, followed by washing with PBS. Four random fields from each membrane were photographed and counted for statistical analysis.

### *In vitro* HUVEC tube network formation assay

For the tube network formation assay, each well of a 96-well plate was pre-coated with 50 μl of Matrigel (BD, USA) and allowed to polymerize for 30 min at 37°C. Next, cells were seeded onto Matrigel-coated wells at a density of 2 × 10^4^ cells per well in conditioned medium at 37°C. Tube formation was found to be optimal after 4 h. Tube images were taken using a digital camera attached to an inverted phase-contrast microscope. Total tube length in each well was measured and calculated using image pro plus (IPP).

### Tumorigenicity *in vivo*

All animal experiments were conducted according to the guidelines of the NJMU Institutional Animal Care and Use Committee. A total of twenty-four nude mice (BALB/c nude mice, Vitalriver, China; four weeks old) were randomly divided into four groups, and SGC7901-NC, SGC-7901-miR-874 inhibitor, AGS-NC or AGS-pre-miR-874 stable cells were inoculated subcutaneously into the flanks of nude mice. The mice were euthanized after 3 weeks.

### Immunohistochemistry (IHC) for subcutaneous grafts

All specimens were fixed in 4% formalin and embedded in paraffin. MaxVision™ techniques (Maixin Bio, China) were used for IHC analysis, according to the manufacturer's instructions. After blocking the endogenous peroxides and proteins, 4 μm sections were incubated overnight at 4°C with diluted primary antibodies specific for STAT3, CD31 (Cell Signaling Technology, USA), and VEGF-A (Abcam, UK). Next, the slides were incubated with an HRP-Polymer-conjugated secondary antibody at 37°C for 1 hour. The slides were then stained with a 3,3-diaminobenzidine solution for 3 min and counterstained with hematoxylin. Tumor slides were examined in a blinded manner. Three fields were selected for examination, and the percentage of positive tumor cells and cell-staining intensity were determined.

### Statistical analysis

Data are expressed as mean ± standard deviation (SD) values. Clinicopathological findings were compared using unpaired *t*-tests or Pearson *x^2^* tests. Analysis of variance (ANOVA) was used to compare the control and treated groups. *P*-values <0.05 were considered to be statistically significant. Statistical analysis was performed using the SPSS software (Version 15.0).

## SUPPLEMENTARY FIGURE


