# Correction: miR-874 functions as a tumor suppressor by inhibiting angiogenesis through STAT3/VEGF-A pathway in gastric cancer

**DOI:** 10.18632/oncotarget.17402

**Published:** 2017-04-24

**Authors:** Xiaoyu Zhang, Jie Tang, Xiaofei Zhi, Kunling Xie, Weizhi Wang, Zheng Li, Yi Zhu, Li Yang, Hao Xu, Zekuan Xu

**Present:** The Acknowledgment information is incomplete.

**Correct:** The proper funding acknowledgments are given below.

Original article: Oncotarget. 2015; 6:1605-1617. doi: 10.18632/oncotarget.2748

