# Supplementary material for: miR-874 functions as a tumor suppressor by inhibiting angiogenesis through STAT3/VEGF-A pathway in gastric cancer
Source: Oncotarget. 2015 Jan 22;6(3):1605–17. doi: 10.18632/oncotarget.2748 (PMC4359318; doi:10.18632/oncotarget.2748)
Supplement: Supplementary file 1 [file oncotarget-06-1605-s001.pdf]

SUPPLEMENTARY FIGURE

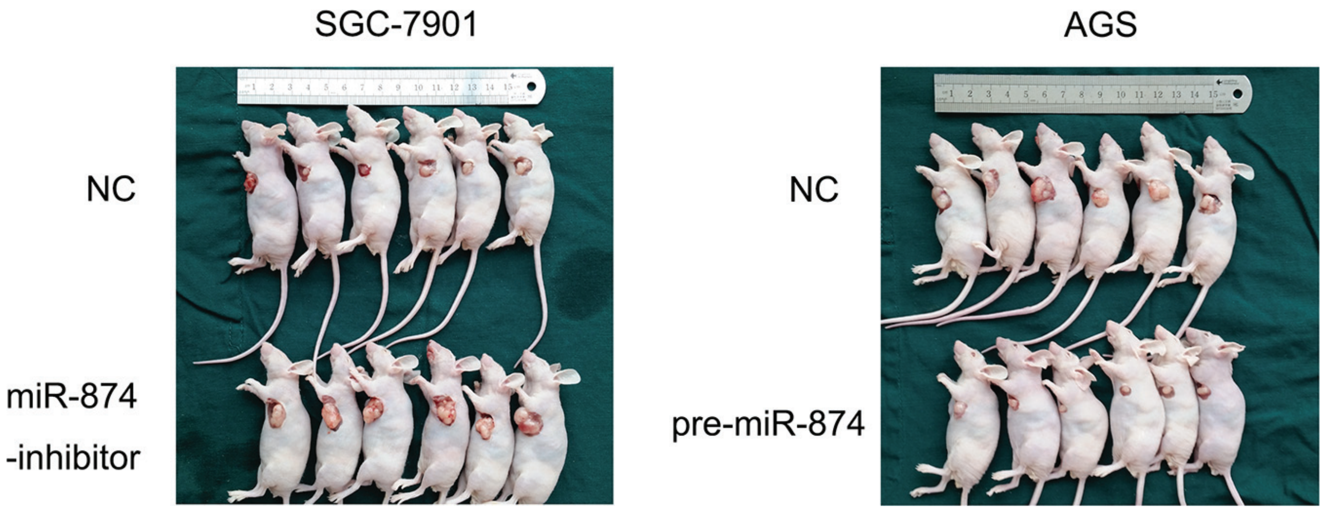

Supplementary Figure 1: Photographs of the nude mice injected with the different groups of cells
